# Generic Properties of Curvature Sensing through Vision and Touch

**DOI:** 10.1155/2013/634168

**Published:** 2013-12-18

**Authors:** Birgitta Dresp-Langley

**Affiliations:** UMR 7357 CNRS, Icube-Unistra, Université de Strasbourg, 67000 Strasbourg, France

## Abstract

Generic properties of curvature representations formed on the basis of vision and touch were examined as a function of mathematical properties of curved objects. Virtual representations of the curves were shown on a computer screen for visual scaling by sighted observers (experiment 1). Their physical counterparts were placed in the two hands of blindfolded and congenitally blind observers for tactile scaling. The psychophysical data show that curvature representations in congenitally blind individuals, who never had any visual experience, and in sighted observers, who rely on vision most of the time, are statistically linked to the same mathematical properties of the curves. The perceived magnitude of object curvature, sensed through either vision or touch, is related by a mathematical power law, with similar exponents for the two sensory modalities, to the aspect ratio of the curves, a scale invariant geometric property. This finding supports biologically motivated models of sensory integration suggesting a universal power law for the adaptive brain control and balance of motor responses to environmental stimuli from any sensory modality.

## 1. Introduction

Interaction of the human body with technological devices relies on the multisensory integration of visual and tactile signals by the human brain, as in the use of global positioning systems for navigation, or the encoding of visual and tactile spatial information for laparoscopic surgery, for example. Experimental studies have shown that the manipulation of visual objects with the two hands and the visual and tactile integration of shape information play an important role in action planning as well as motor control [1, 2]. This is particularly important in spatial perception by the blind who never had visual experience (congenitally blind people) but who are nonetheless perfectly capable of understanding the physical environments which surround them and of forming exact representations of complex spatial geometry. Visual and tactile representations of space are thus likely to share common generic properties. Understanding how the blind compensate through touch for lack of visual data is relevant in rehabilitation research and for the effective design of technological aids designed to help the blind explore real-world spaces [3]. Moreover, knowing how visual and tactile sensing is interactively programmed in the human brain has implications for medical robotics and clinical neurology as, for example, the study and treatment of neurological disorders such as spatial neglect [4, 5] or tactile allodynia [6]. Physical and perceptual models, tested in the light of statistical probabilities, are needed to extend our knowledge on how visual and tactile brain representations function, how they interact, and what they have in common.

The unified brain processing of signals mediated by vision and touch involves cortical neurons with nonclassic receptive field structures, functionally identified in the monkey brain [7–11]. The receptive fields of such multimodal neurons correspond to action spaces that are consistent with the early theories of active perception proposed by Gibson [12, 13], who was among the first to suggest that visual and tactile representations may share common generic properties. The sensorial pathways for visual and tactile processing are independent at early stages of encoding. At later stages, a coupling of signals must take place in the brain to enable sensory coordination. This inevitably involves feedback signals from motor responses. It is likely that power laws, known to govern the integration of chemosensory signals in modality specific pathways, reflect the generic mechanism that enables the invariant control and balance of brain activities related to the processing of signals from different sensory pathways. Yet, model hypotheses backed by consistent psychophysical data are still lacking. In this study, we looked for generic properties of spatial representations formed on the basis of vision and touch related to the mathematical properties of curved objects, represented on a computer screen for visual exploration, and their physical counterparts placed in the two hands of blindfolded and congenitally blind observers for tactile exploration.

Curvature is a mathematical property of the physical world that is of considerable importance in complex system science. Curvature guides physical, chemical, and biological processes, like protein folding, membrane binding, and other biophysical transformations [14]. The representation and cognition of curvature starts at the biochemical level of living organisms capable of sensing this property in their near or distant physical environments [15]. Curvature is also a perceptual property extracted from physical stimuli to form veridical representations in highly developed organisms such as the brain. The ability of processing curvature visually is highly developed in humans. The spatial precision with which man can distinguish a curve from a straight line reflects only a fifth of the physical spacing between two neighboring visual receptors and a tenth of the smallest receptive field centre of ganglion cells found in the primate retina [16, 17]. Among the geometric properties of curves exploited by the brain to achieve such an astonishing performance are the distance between the line or chord that joins the two ends of a curve and the parallel line tangent to that curve, the sagitta [18], and the ratio between the sagitta and the length of the chord, also called aspect ratio [19]. Visual images of curves with the same aspect ratio but varying sagitta produce identical detection thresholds, suggesting that the visual processing of curves may well be independent of scale. The aspect ratio of a curve is a scale-invariant parameter which conveys a global representation of the spatial extent or area covered by the curve, while the sagitta provides a strictly local cue to the point of maximum curvature. A mathematical model for planar curve generation based on vertically and horizontally oriented ellipses was found to produce the most reliable psychometric functions for visually mediated estimates of curvature as a function of the aspect ratio of a large number of planar curves, presented in random order on a computer screen to human observers [20]. The stimuli used in these visual experiments were designed to reflect properties of real-world objects that can be manipulated with the hands.

**Figure 1:**
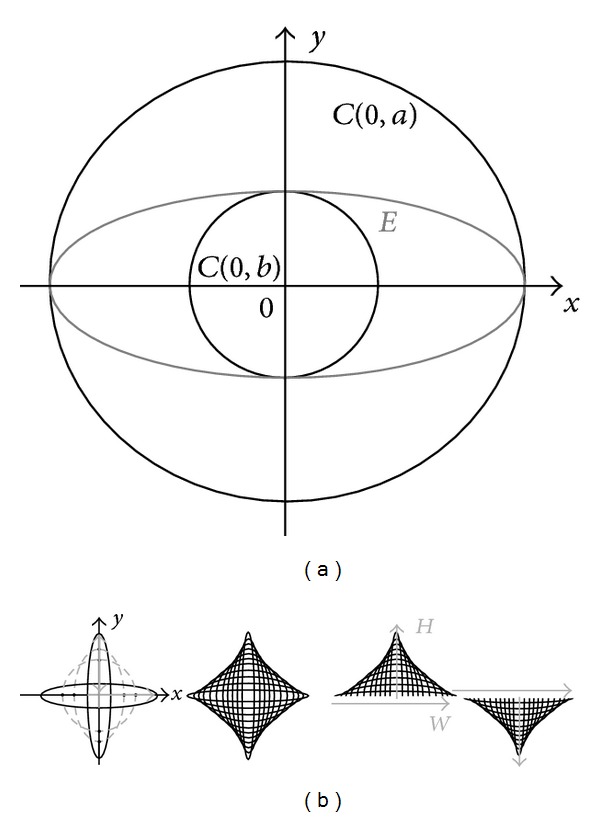
Ellipses were generated in AUTOCAD following the principle of planar projection of ellipses by affinity with concentric circles (a). The major and minor axes of symmetry of an ellipse *E* determine sagitta, or maximum height (*H*), and chordlength, or width (*W*), of the curved contour of half that ellipse. For this study, the ellipses were cut in half at an axis of symmetry (b) to generate curve stimuli with varying orientation (upward and downward), height (*H*), and width (*W*).

The most parsimonious mathematical definition of curvature relates to circles and ellipses. In terms of geometry, curves derived from circles and ellipses share certain properties, with the circle being a particular case of the ellipse. Also, the choice of elliptic curves for studying perceptual mechanisms is biologically motivated given their symmetry and the observation that, in the real world, the curvature of the contours of natural objects corresponds to a wide range of symmetrical shapes with the Euclidean properties of ellipses. For the purpose of this study, we used projective geometry to generate curves from ellipses, by affinity with concentric circles (Figure 1), as will be explained in the next section.

## 2. Material and Methods

**Figure 2:**
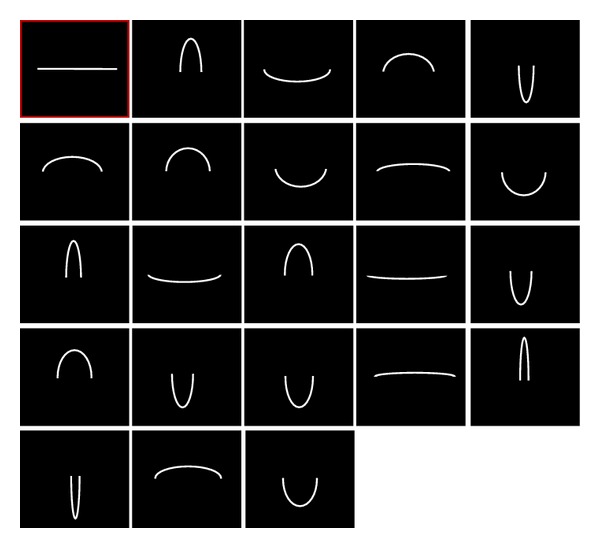
Computer generated (AUTOCAD) curve stimuli for the visual experiment, presented in random order on a computer screen. Curve orientation (upward or downward) varied randomly. All curves were presented at high contrast.

**Figure 3:**
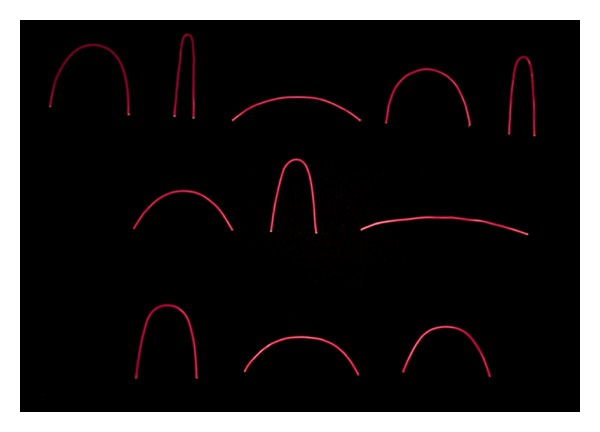
A photograph of the veridical real-world counterparts of the virtual curves, with identical *H* and *W* (in centimetres), used in the tactile experiment. As in the visual experiment, curves were presented in random order and curve orientation (curve placed into the two hands of an observer with upward and downward orientation) also varied randomly. Only upward orientation is shown here in this photograph.

Virtual curves and their real-world counterparts were presented to human observers for psychophysical scaling in two separate experiments. One experiment consisted of a randomly presented sequence of virtual curves presented as visual stimuli on a computer screen (Figure 2) to observers with normal vision. In the other experiment, the real-world counterparts of these curves (Figure 3) were presented manually, in random order and according to the same procedure, to blindfolded and congenitally blind observers. In both tasks, observers had to rate the perceived magnitude of each stimulus on a standard psychophysical scale [21, 22] with values ranging from 0 for no curvature perceived to 10 for maximum curvature perceived. This classic psychophysical scaling procedure has proven a reliable tool for studying perceptual sensations and their internal representation. Psychophysical scaling aims at linking psychological and physiological mechanisms to the physical or mathematical properties of the outside world and was introduced at the beginning of the last century by eminent medical scientists such as Fechner and Wundt. The method is now widely applied in contemporary medical research and clinical testing.

### 2.1. Mathematical Properties of the Curves

The curves for this study were derived from planar ellipses, generated in AUTOCAD on the basis of projective geometry, through transformation by affinity with concentric circles (Figure 1(a)), a procedure frequently used in digital rendering and design. To understand how ellipses are obtained in such a way, it is useful to recall some of the properties of concentric circles, which share the same centre. In the Cartesian plane, the principal circle with centre 0 (*C*_0,*a*_) is defined in terms of

(1) $$\begin{array}{c} R^{2}\left(C_{0,a}\right)={\left(x\right)}^{2}+{\left(y\right)}^{2}, \end{array}$$

where *R* is the radius of the circle and *x* and *y* the two-dimensional spatial coordinates of the points falling on its perimeter. The second, concentric circle is obtained from the first one by

(2) $$\begin{array}{c} R^{2}\left(C_{0,b}\right)={\left(x+\delta x\right)}^{2}+{\left(y+\delta y\right)}^{2} \end{array}$$

or

(3) $$\begin{array}{c} R^{2}\left(C_{0,b}\right)={\left(x-\delta x\right)}^{2}+{\left(y-\delta y\right)}^{2}. \end{array}$$

Ellipses as projected images of concentric circles (Figure 1(a)) may be defined in terms of

(4) $$\begin{array}{c} \left(x,y\right)=\left(bx,ay\right) \end{array}$$

of the principal circle *C*(0, *a*) and

(5) $$\begin{array}{c} \left(x,y\right)=\left(\left(\frac{a}{b}\right)x,y\right) \end{array}$$

of the secondary circle *C*(0, *b*). This transform is sometimes referred to as a particular case of Newton's transform [23]. In the Cartesian plane, the ellipse (*E*) is defined in terms of

(6) $$\begin{array}{c} E=\frac{x^{2}}{a^{2}}+\frac{y^{2}}{b^{2}}=1, \end{array}$$

with axes *a* and *b* being the axes of symmetry intersecting at its center (Figure 1(a)). The larger axis of the two (*a*) is referred to as the major and the smaller (*b*) as the minor. The majors and the minors are directly linked to the sagitta, or maximum height (*H*), and the chordlength, or width (*W*), of the curves that were derived from the ellipses here (Figure 1(b)), which were cut in half at one of the axes of symmetry to generate curves with varying orientation in the plane (upward or downward), varying height (*H*), and varying width (*W*). The numerical values (in centimetres) of these curve parameters are given in Table 1.

### 2.2. Virtual Curves for Visual Presentation

Curves for visual presentation (Figure 2) generated in AUTOCAD by a group of first-year design students were stored as individual images in an image library for the experiment, which was run on an IBM Pentium III equipped with a standard colour screen. The size of a single pixel on the screen corresponded to 0.0025 cm. The physical curve parameters of maximum height (*H*) and width (*W*), given in centimetres here in Table 1, translate into degrees of visual angle (Deg *V*), which reflects the size of a given parameter as it is perceived by the human eye as a function of the distance of the observer from the object viewed, using the linear transform,

(7) $$\begin{array}{c} \text{Deg }V=2\arctan\left(\frac{S}{2D}\right), \end{array}$$

where *S* is the physical stimulus parameter (in centimetres) and *D* the viewing distance (also in centimetres) or distance of the observer's head from the screen, held constant at 120 centimetres in this experiment. All stimuli were presented foveally, bearing in mind that the diameter of foveal vision is limited to approximately 5 degrees of visual angle.

### 2.3. Real-World Curves for Tactile Exploration

The real-world counterparts of the curves for the tactile experiments were designed by the same group of design students. Ellipses with the same height and width properties as those generated in AUTOCAD to build a visual library of curve images presented for presentation on a computer screen were drawn on cardboard with compass and ruler following a method for drawing ellipses manually (the “gardener's ellipse method,” described on http://www.mathopenref.com/). The curves, made of flexible plastic-coated wire cable (Figure 3), were bent by hand and matched visually to the images of the half ellipses. The cable material was flexible but rigid enough for a given curve once formed to retain its shape reliably under gentle manipulation with the fingers of the two hands.

### 2.4. Participants

Six men and four women, between 25 and 32 years old and equipped with normal vision, participated in the visual experiment. Five women and three men, between 26 and 48 years old and equipped with normal vision but blindfolded for the purpose of the experiment, and two congenitally blind observers, both men, participated in the tactile experiment. Each of the individuals only participated in one of the two experiments, which were conducted in accordance with the Declaration of Helsinki (1964). None of the participants was aware of the aims of the study.

### 2.5. Procedure

Eleven curves with positive curvature (upward orientation) and eleven curves with negative curvature (downward orientation) in the two-dimensional plane were presented on a computer screen in the visual experiment. The stimuli were presented in random order in 22 successive trials. Each curve was presented once for two seconds in a session. Curve orientation (upward or downward) also varied randomly. All curves were presented at high contrast, defined by a contour segment of the thickness of a single pixel on the screen with a luminance of 40 cd/m^2^. The luminance of the screen background on which the curves were presented was 2 cd/m^2^. Observers were seated comfortably in a semidark room, with their heads resting on a head-and-chin rest at a distance of 120 centimetres from the computer screen. Their hands rested on a desk with a computer keyboard, which they had to use to indicate the perceived magnitude of curvature for each stimulus by typing a number from zero to ten on the computer keyboard. Typing the “enter” key then triggered presentation of the next stimulus in the visual experiment. Observers were instructed that they were going to view a series of curves, one at a time, and were asked to produce a number between 0 and 10 that was to reflect the intensity (magnitude) of curvature they spontaneously perceive when a given curve comes up on the screen. A straight line was shown at the beginning of each individual session, solely to make sure that the observer spontaneously typed “0” on the computer keyboard and had, indeed, understood the instruction to scale curvature. Psychophysical scaling does not require giving lower and upper limits of a physical stimulus to a healthy adult human observer. Once fully developed, the nonpathological human brain is capable of reliably scaling any stimulus on the basis of internally represented (learnt) upper and lower limits [21]. In the tactile experiment, observers were instructed that they were going to explore a series of curved cables, one at a time, with the fingers of their two hands. They were asked to produce a number between 0 and 10 that was to reflect the intensity (magnitude) of curvature they perceive when a given curve is placed in their hands and to explore each curve very gently without pulling or bending the wire cables. An experimenter was present to make sure that this instruction was adhered to and that none of the observers deformed a cable. A perfectly straight wire cable was placed in the two hands of an observer at the beginning of the trials to make sure that each of them spontaneously replied “zero curvature” and that the instruction to scale curvature had been understood. The stimuli were given to the observers, in random order, one at a time. Each of the eleven curves was presented to each observer twice, once pointing upward and once pointing downward, generating a sequence of 22 successive trials as in the visual experiment.

## 3. Results and Discussion

The data from the two experiments were analyzed in terms of average sensations of visually and haptically (exploration with the two hands) perceived curvature (average subjective magnitudes and their standard deviations) as a function of the local curve parameter sagitta, or height of the curve (*H*), and as a function of the scale invariant parameter aspect ratio (*H*/*W*). These data were then subjected to mathematical modelling and compared across the two sensory modalities to examine whether common characteristics are found.

### 3.1. Vision

**Figure 4:**
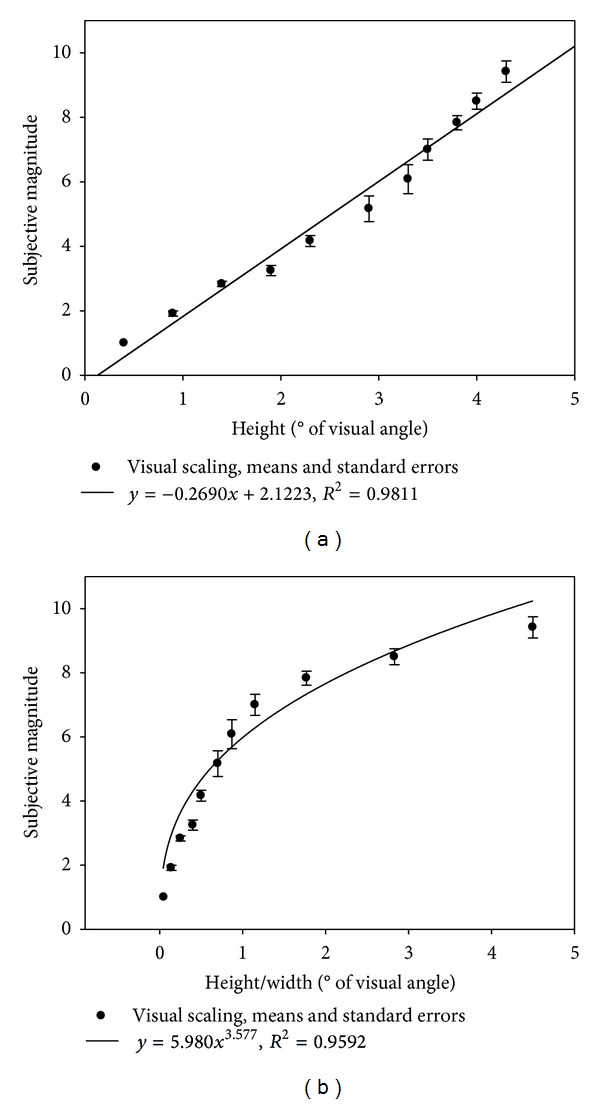
Visually perceived magnitude of curvature as a function of the height (*H*), expressed in degrees of visual angle (a), and as a function of their aspect ratio (*H*/*W*), a scale invariant parameter (b) of the curves, presented on a computer screen for visual scaling in the first experiment.

Visually perceived curvature was found to increase linearly with the local parameter sagitta, or height (*H*) of the curve (Figure 4(a)). The goodness of the linear fit is satisfactory, as indicated by a linear regression coefficient (*R* ^2^) of 0.98. This finding is entirely consistent with previous data [20] showing that sensations of curvature in the visual modality are a linear function of the sagitta. When plotted as a function of the aspect ratio (*H*/*W*), average visual magnitude of curvature is found to increase with the aspect ratio according to a power law (Figure 4(b)), with an exponent of 3.57 and a correlation coefficient (*R* ^2^) of 0.96. The goodness of the power fit is statistically significant (*F*(1,9) = 211.86, *P* < 0.001).

### 3.2. Touch

**Figure 5:**
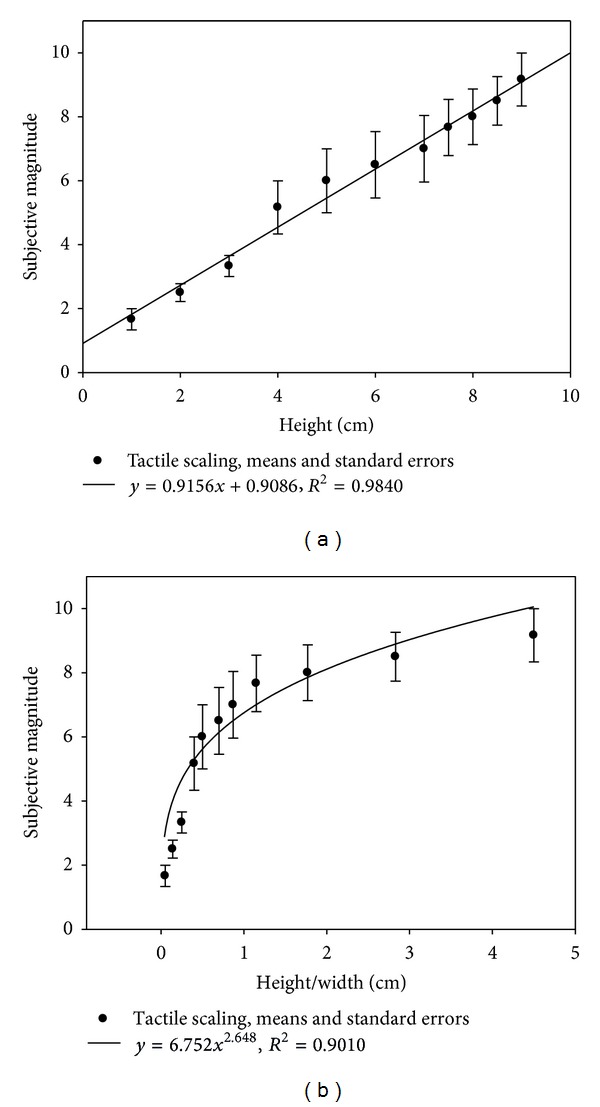
Haptically sensed magnitude of curvature as a function of the height (*H*), expressed in centimeters (a), and as a function of the scale invariant aspect ratio (*H*/*W*) of the curves (b) explored with two hands by blindfolded observers in the second experiment.

**Figure 6:**
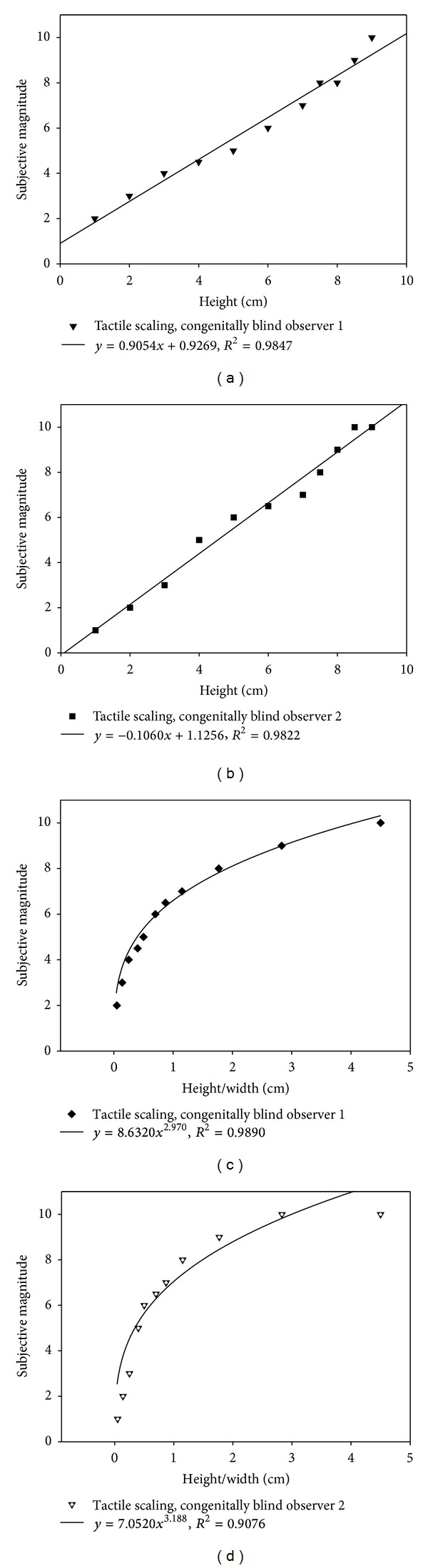
Haptically sensed magnitude of curvature as a function of the height (*H*), expressed in centimeters ((a), (b)), and as a function of the scale-invariant aspect ratio (*H*/*W*) of the curves ((c), (d)) explored with two hands by congenitally blind observers in the second experiment.

Haptically perceived curvature by seeing individuals who were blindfolded and could not see the curves and rely mostly on their vision in everyday life was, like visual curvature, found to increase linearly with the local parameter *H* (Figure 5(a)). The goodness of the linear fit to the tactile data was as for the visual data, with a linear regression coefficient (*R* ^2^) of 0.98. When plotted as a function of the aspect ratio (*H*/*W*), average haptically sensed magnitude of curvature is, like visually perceived curvature, found to increase with the aspect ratio according to a power law (Figure 5(b)), with an exponent of 2.6 and a correlation coefficient (*R* ^2^) of 0.90. The goodness of the power fit is statistically significant (*F*(1,9) = 81.91, *P* < 0.001). Curvature scaled through touch by congenitally blind individuals, who never had visual experience and rely mostly on their other senses in everyday life, was also found to increase linearly with *H*. Analysis of the individual data of each of them (Figures 6(a) and 6(b)) shows that the goodness of the linear fits to the data of blind observers is excellent, with regression coefficients (*R* ^2^) of 0.95 and 0.98. When plotted as a function of the aspect ratio (*H*/*W*), magnitude of curvature haptically sensed by the congenitally blind observers is, again, found to increase with the aspect ratio according to a power law (Figures 6(c) and 6(d)). The goodness of the power fits is statistically significant for both observers (*F*(1,9) = 809.35, *P* < 0.001 and *F*(1,9) = 88.41, *P* < 0.001). The exponents of the power functions are similar (2.97 and 3.19). Whether such statistically robust data can be expected from any congenitally blind patient will largely depend on their ability to perform the psychophysical scaling task and whether other cognitive impairments are diagnosed. We consider the two, otherwise healthy, patients tested here in this experiment as representatives of a larger population of, otherwise healthy, congenitally blind patients.

The results demonstrate that symmetric curvature is consistently scaled by the human brain on the basis of internal representations. These internal representations could be, as the results from this study here would suggest, statistically invariant when derived from either visual or tactile sensations. Such internal scaling of the spatial property of curvature would be similar to the psychophysical scaling of intensities of sensory stimuli. Earlier studies [21, 22] have demonstrated quite comprehensively that in sensory modalities such as vision, audition, olfaction, or taste, the perceived intensity of a stimulus obeys a power law, with exponents similar to those found here in our study. Physiological data and recent biologically motivated mathematical models point towards the functional significance of power laws in neural pathways where they were found to govern mechanisms through which sensory feedback signals enable the brain to adaptively control and balance motor responses to physical stimuli.

## 4. Conclusions

The findings from this study show that curvature representations in congenitally blind individuals, who never had any visual experience, and in seeing observers, who take in the physical world through their visual systems most of the time, are statistically linked to the same mathematical properties [23–25] of curved objects, whether these are sensed visually on the basis of virtual representations on a computer screen or directly by the two hands on the basis of real-world objects. This rather novel finding supports the hypothesis that cognitive representations derived from vision and touch have common generic properties [3, 12, 13] that can be exploited for the study and design of technological devices to aid blind individuals to compensate for the lacking visual brain signals.

More importantly, it is shown here that the perceived magnitude of object curvature, sensed through either vision or touch, is linked by the same power law, with similar exponents for the two sensory modalities, to a scale invariant mathematical property of the physical stimuli. This is consistent with the idea that a unified representation of tactile and visual signals in the brain would necessarily involve scale invariant mechanisms at some stage of processing. The similarity of the findings reported here with earlier psychophysical data on chemosensory signal processing in the brain leads to conclude that chemical and tactile signals are at some stage integrated with common mechanisms in the brain. The power model suggested here supports other biologically motivated models of sensory integration suggesting a universal power law for the adaptive brain control and balance of motor responses [26] to chemical as well as tactile or mechanical stimuli.

## Figures and Tables

**Table 1:** Mathematical parameters of the eleven curves (stimuli) in terms of width (*W*), sagitta (*H*), expressed in centimetres, and the scale-invariant aspect ratio (*H*/*W*), as defined here in the introduction. Parameters of virtual curves presented on a computer screen to one group (visual experiment) and their real-world counterparts used for tactile sensing by another group of blindfolded or congenitally blind observers (tactile experiment) were the same.

*W*	*H*	*H*/*W*
2 cm	9 cm	4.50
3 cm	8.5 cm	2.83
4.5 cm	8 cm	1.77
6.5 cm	7.5 cm	1.15
8 cm	7 cm	0.87
8.5 cm	6 cm	0.70
9 cm	5 cm	0.50
10 cm	4 cm	0.40
12 cm	3 cm	0.25
14 cm	2 cm	0.14
18 cm	1 cm	0.05
